# Selective Enrichment and Sequencing of Whole Mitochondrial Genomes in the Presence of Nuclear Encoded Mitochondrial Pseudogenes (Numts)

**DOI:** 10.1371/journal.pone.0037142

**Published:** 2012-05-14

**Authors:** Jonci N. Wolff, Deborah C. A. Shearman, Rob C. Brooks, John W. O. Ballard

**Affiliations:** 1 School of Biotechnology and Biomolecular Sciences, University of New South Wales, Sydney, Australia; 2 Evolution and Ecology Research Centre, University of New South Wales, Sydney, Australia; 3 School of Biological, Earth and Environmental Sciences, University of New South Wales, Sydney, Australia; Ben-Gurion University of the Negev, Israel

## Abstract

Numts are an integral component of many eukaryote genomes offering a snapshot of the evolutionary process that led from the incorporation of an α-proteobacterium into a larger eukaryotic cell some 1.8 billion years ago. Although numt sequence can be harnessed as molecular marker, these sequences often remain unidentified and are mistaken for genuine mtDNA leading to erroneous interpretation of mtDNA data sets. It is therefore indispensable that during the process of amplifying and sequencing mitochondrial genes, preventive measures are taken to ensure the exclusion of numts to guarantee the recovery of genuine mtDNA. This applies to mtDNA analyses in general but especially to studies where mtDNAs are sequenced *de novo* as the launch pad for subsequent mtDNA-based research. By using a combination of dilution series and nested rolling circle amplification (RCA), we present a novel strategy to selectively amplify mtDNA and exclude the amplification of numt sequence. We have successfully applied this strategy to *de novo* sequence the mtDNA of the Black Field Cricket *Teleogryllus commodus*, a species known to contain numts. Aligning our assembled sequence to the reference genome of *Teleogryllus emma* (GenBank EU557269.1) led to the identification of a numt sequence in the reference sequence. This unexpected result further highlights the need of a reliable and accessible strategy to eliminate this source of error.

## Introduction

The incorporation of an α-proteobacterium into a larger eukaryotic cell some 1.8 billion years ago is regarded as one of the cornerstones of eukaryotic evolution [1]. The engulfed bacterium was reduced over evolutionary time to an organelle, assumed to be central to the rise of eukaryotic diversity by awarding its host the capacity of oxidative phosphorylation: the mitochondrion. Initially, the engulfed organism may have harboured several thousand genes, similar to that of present-day gram-negative bacteria of the genus Rickettsia. Measuring several megabases (Mb), sizes of present-day α-proteobacterial genomes stand in stark contrast to those of animal mitochondrial genomes, which are typically 16 kb in size with highly conserved gene content [2], [3], [4].

The discrepancy in gene content between present-day α-proteobacteria and mitochondrial genomes is the product of intracellular horizontal gene transfer during which the vast majority of the bacterial gene content was transferred to the host nucleus, with their gene product reimported into the organelle. Although presumably significantly reduced, studies in the murine model estimate the mitochondrial proteome at 940 proteins with the overwhelming majority of these proteins inevitably encoded in the nucleus [5]. In theory, for genes elemental to either host or endosymbiont which have been transferred during organellar genome reduction, at least two functional copies must have existed at some stage: one in the nucleus and one in the developing organelle, to maintain metabolic functionality. The gain of functionality of nuclear copies rendered their mitochondrial counterparts redundant and is hypothesized to have led to their deletion in the organelle. Step-wise reduction in gene content and with that a progressive loss of independence ultimately turned the former free-living bacterium into an organelle.

As an inevitable consequence of this gene transfer additional copies of mitochondrial genes can be found in the nucleus reflecting a snapshot of this process. These DNA sequences, generally referred to as numts (nuclear encoded mitochondrial DNA [6]), are generally non-functional but of high similarity to their *bona fide* counterparts and can range in length from a few bases to entire copies of mitochondrial genomes [7], [8]. Whereas the potential contamination of mitochondrial DNA (mtDNA) data sets with numts was regarded as somewhat exotic a decade ago, it is now assumed a constant threat to studies harnessing the advantages of mtDNA as a molecular marker. Genome-wide BLAST searches have revealed that numts are widespread among animal species but not equally abundant [8]. The fully sequenced genome of the Gray Short-tailed Opossum, for example, hosts a staggering 2 Mb worth of numt sequence whereas similar BLAST searches failed to detect any such sequences in the zebrafish [8], [9]. Even closely-related species can vary significantly with *Caenorhabditis briggsae* revealing 14 Kb of numt sequence and *Caenorhabditis elegans* a mere 100 bp [9]. What drives these differences is not known but a positive correlation between genome size and numt content has been put forward as major force of inter-species inequalities [9]. Whereas this intracellular gene transfer appears to be slowing down in animals, it is still a much more dynamic process in plants where dual transcription of mitochondrial and nuclear copies of single genes has been documented in a variety of species [10].

**Figure 1:**
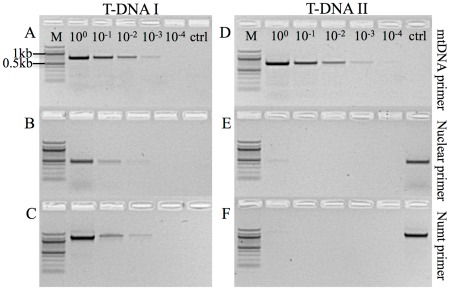
Test for the presence of mitochondrial and nuclear DNA in serial dilution series' T-DNA I & T-DNA II. T-DNA I was used as template in A–C and T-DNA II in D–F. The highest dilution step in which the presence of nuclear DNA was revealed using both nuclear and numt-specific primers was step 3 (10^−3^, B, C) and step 4 (10^−4^) for mtDNA primers (A) on T-DNA I. For T-DNA II, dilution step 1 (10^0^) was the highest dilution showing residual nuclear and numt amplification and step 5 (10^−4^) for mtDNA. Controls (ctrl) are no template controls in A–D and positive controls in E–F. M: DNA ladder (100 bp).

Owing to their origin, numt sequences are inevitably highly similar and generally retain close homology to their mitochondrial counterparts (with recent numts revealing higher resemblance than ancient ones) and can therefore easily be confused with one another. Slight sequence difference between closely related species can lead to the amplification of mitochondrial sequences in one species and to the preferential amplification of numt sequences in another species [11]. While numts can offer an additional source of genetic information employable to trace evolutionary relationships [12], [13], their unnoticed presence in mitochondrial data sets poses a substantial threat to accurate interpretation of data sets and may consequently lead, if remaining unidentified, to incorrect conclusions [14], [15].

**Figure 2:**
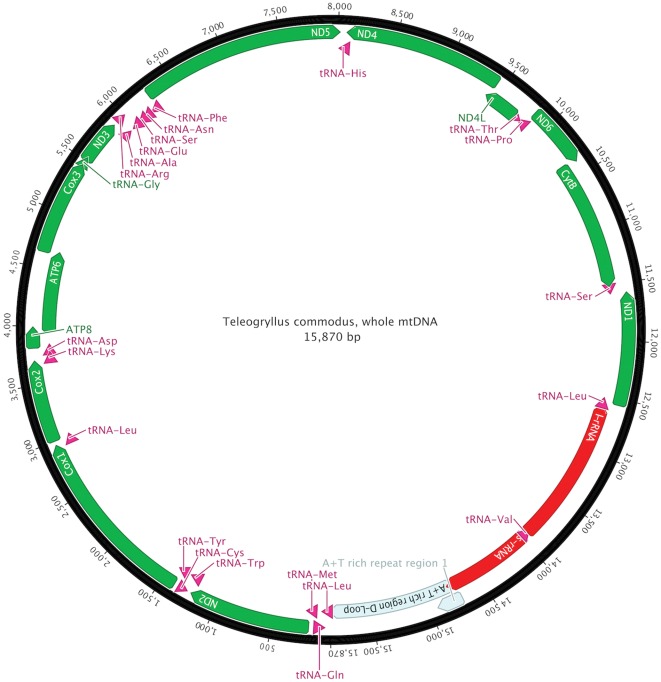
Schematic illustration of the mitochondrial genome of *T. commodus*.

A variety of strategies have been suggested to evade the unintentional co-amplification of numts: (i) using cDNA as template for amplification of mtDNA, assuming numts are non-functional and generally not transcribed [16], [17]; (ii) basic sedimentation of mitochondria before DNA extraction at low speed or the more elaborate separation of circular (mtDNA) and linear DNA (nuclear DNA; nuDNA) after DNA extraction at ultrahigh speed [18], [19]; (iii) amplification via long-distance and nested PCR, assuming numt sequences are generally short [20], [21]; (iv) exclusion of nuDNA via dilution of DNA templates assuming a higher copy number for the mitochondrial than the nuclear genome in whole genomic DNA extracts [21]. Although previously applied successfully to extract and/or amplify mtDNA, these approaches are either labour-intensive or lack global applicability, failing at times to prevent the co-amplification of numts.

In this manuscript we present a simple, time- and cost-effective strategy virtually nullifying the threat of contaminating numt sequences. This strategy is applicable to any species and requires only minute amounts of tissue. It consists of a combination of standard DNA extraction, serial dilutions and a nested rolling circle amplification (RCA). We developed and applied this strategy during the *de novo* sequencing the mitochondrial genome of the Black Field Cricket (*Teleogryllus commodus*) after co-amplifying numts using a combination of conserved and newly designed primers ([22], Figure S1). Accurate *de novo* sequencing of mitochondrial genomes is of utmost importance as these genomes generally serve as initiator and foundation for subsequent mtDNA-based research. Sequencing the mitochondrial genome of the Black Field Cricket is ideal to test our strategy as many orthoptera are known to harbour large genomes and high numbers of numt sequences [23], [24].

## Methods

### Ethics statement

Ethics Approval was received from the UNSW Animal Care and Ethics Committee (Approval ID 10/72B).

### Selective amplification of mtDNA

DNA extraction and Serial Template Dilution I (T-DNA I): DNA was extracted from 1 mg of muscle tissue of the hind leg using the Gentra Puregene® Cell Kit (Qiagen, Hilden, Germany). Extractions were incubated with 1.5 µL of 20 mg mL^−1^ Proteinase K at 55°C for two hours and vortexed every 20 minutes. Extracted DNA was used to prepare a ten-fold dilution series (10^0^ to 10^−4^) in TE buffer (10 mM Tris, 1 mM EDTA, pH 8.0).

Standard PCR and Template identification for RCA: Standard PCR was used to identify the dilution step (T-DNA I) adequate for rolling circle amplification, *i.e.* the DNA dilution that resulted in no measurable or residual amplification using nuclear primers but strong amplification using mitochondrial primers. PCR reactions were carried out in 25 µL reactions containing 1 µL of diluted DNA, 5× Crimson buffer, 1.5 mM MgCl_2_, 0.2 mM dNTPs, 0.5 U Crimson® Taq (New England BioLabs® Inc., Ipswich, USA) and 10 pmol of forward and reverse primer. Three primer pairs were used: one pair preferentially amplifying numt sequence (2510F×TK-N3796, 1286 bp; [25], Table S1), a nuclear pair (HexF×HexR, 419 bp; [26]) and primers known to amplify mtDNA only (TK-J3990×A6-N4563, 749 bp; [25], [26]). PCRs were run on a Bio-Rad® Dyad Peltier Thermal Cycler (Bio-Rad® Laboratories, Hercules, USA). PCR profile: denaturation at 96°C for 2 min; denaturation at 96°C for 20 sec; annealing at 50°C for 15 sec; extension for 1∶15 min at 68°C; 32 cycles.

Rolling Circle PCR and Serial Template Dilution II (T-DNA II): RCA was carried out using the REPLI-g Mitochondrial DNA Kit (Qiagen, Hilden, Germany) with 1 µL template identified to reveal no significant amplification using nuclear primers according to the manufacturer's recommendations. Incubation time was extended to 12 hours and 9 additional universal and cricket-specific primers (0.25 µL of 2 µM solution per primer; primers: TM-J210, C1-J2195, 7174F, 12520F, C1-2776, A6-N4563, C3-N5460, 12218R, 13461R, see [25], Table S1) were added to increase the specificity of the reaction and to increase absolute yield. Amplified DNA was used to prepare a ten-fold dilution series (10^0^ to 10^−4^) in TE buffer.

### *De novo* amplification and sequencing of the mitochondrial genome

Amplification and subsequent sequencing was conducted on T-DNA II using the highest dilution still producing a strong PCR signal using mtDNA primers but no detectable amplification using nuclear primers. Primers for standard PCR were the same as those for sequencing and were designed using the *Teleogryllus emma* sequence (GenBank EU557269.1; Table S1). PCR followed standard thermal profiles (see above) with an annealing temperature of 57°C for all primer combinations. Amplicons were purified using ExoSAP-IT® (USB Amersham, Buckinghamshire, UK) to remove primers and unincorporated dNTPs following the manufacturer's recommendations. Sequencing was performed using the purified amplicon using the BigDye terminator v3.1 kit (Applied Biosystems Inc., Foster City, USA). Reactions were carried out in 10 µL reactions containing 2 µL purified PCR product and 3 pmol primer according to the manufacturer's recommendations and analysed at the Ramaciotti Centre for Gene Function Analysis (University of New South Wales, Sydney, Australia) on an ABI-3730 DNA Sequencer (Applied Biosystems Inc., Foster City, CA, USA). PCR and sequencing reactions were duplicated to confirm sequence accuracy.

### *In silico* analysis

Sequences were analysed, aligned, assembled and illustrated using Geneious [27] and the mtDNA of *T. emma* (GenBank version EU557269.1) as reference. Protein-coding genes, ribosomal subunits and tRNAs were identified by alignment in accordance with the annotated genome of *T. emma* and further confirmed by multi-species comparisons using NCBI-BLAST [28]. Further, functionality of tRNAs was tested by confirming correct secondary structures and anticodons using tRNAscan-SE 1.21 [29]. The large and small ribosomal subunits were extended to the boundary of flanking genes where appropriate. Sliding window analysis was conducted using DnaSP [30], comparative sequence analysis using Geneious [27], and to compare amino acid sequence and secondary structure consistency between multiple species we used the online suite PRALINE [31].

Sliding window analysis detected a region of divergence between *T. commodus and T. emma* around the ND3 gene. To investigate this result further ten insect ND3 sequences were retrieved from NCBI (http://blast.ncbi.nlm.nih.gov/). ND3 sequences from 6 additional ensifera (*Elimaea cheni*, NC_014289.1; *Deracanthi onos*, NC_011813.1; *Conocephalus maculatus*, NC_016696.1; *Troglophilus neglectus*, NC_011306.1; *Gryllotalpa pluvialis*, NC_011302.1; *Myrmecophilus manni*, NC_011301.1), one coleptera (*Anoplophora glabripennis*, DQ768215.1) and three diptera (*Drosophila melanogaster*, NC_001709.1; *Anopheles darling*, NC_014275.1; *Culex pipiens pipiens*, NC_015079.1) were retrieved.

### Test of general applicability

To test the applicability of this strategy to samples other than cricket tissue samples used here, we have applied this strategy to a genomic DNA sample extracted from whole blood of the Australian Dingo. DNA was extracted using the DNeasy Blood & Tissue Kit (Qiagen, Hilden, Germany) and the two fragments amplified were a 722 bp region of the mitochondrial D-loop according to Savolainen et al. [32] and a 396 bp region of the nuclear *CBD103* gene according to Anderson et al. [33]. T-DNA I and T-DNA II were produced as described above.

## Results

To exclude contamination of our dataset with numts during *de novo* sequencing of the Black Field Cricket mtDNA, we prepared a ten-fold dilution series of the DNA extract to determine the DNA concentration revealing good amplification using mtDNA primers and only residual amplification at most of nuclear DNA (Figure 1). Consequently, dilution step 3 (10^−3^) was identified as suitable level of dilution for template DNA for subsequent RCA. We then prepared another ten-fold dilution series using the RCA product and used this as template for standard PCR using both mtDNA and nuclear primers to control for the presence of residual nuclear DNA and to confirm the selective enrichment of mtDNA (Figure 1). To test the general applicability of the selective enrichment of mtDNA, this strategy was also employed successfully on a whole genomic DNA extract from mammalian blood (Figure S5).

Although dilution 2 (10^−1^) of T-DNA II showed no presence of nuclear DNA, the highest dilution still producing a strong signal using mitochondrial primers was used (step 3, 10^−2^) for subsequent PCR and *de novo* sequencing to minimise the potential presence of residual nuDNA (Figure 1 D–F). We used 14 primer pairs producing partially overlapping fragments with an average length of 1.3 kb. Sequence assembly resulted in a circular molecule 15,870 bp in length, containing 37 genes (GenBank JQ686193.1, Figure 2). Typical for animal mitochondrial genomes, 13 of these genes are protein-coding (subunits of the electron transport chain), 22 are tRNAs and two encode the small and large subunit of ribosomal rRNA (Figure 2). At 26.2%, the G/C content of *T. commodus* is similar to that of *T. emma* (26.5%), the closest related species for which the whole mitochondrial genome sequence is available. The crickets share 13,811 identical sites, or 86.5% sequence homology and reveal the same gene order. We used sliding window analysis to detect regions of high divergence (Figure S2). The highest variability was detected in the region between nucleotide positions 5457 and 5853 (in reference to GenBank EU557269.1). This region encodes the ND3 gene and partial sequences of flanking tRNAs either side of ND3.

Owing to the extent of sequence difference between the two sequences in this region we extracted the ND3 sequence and subjected it to further analysis. An initial BLAST search of both sequences performed on public databases resulted in multiple hits highly similar to the newly sequenced ND3 sequence of *T. commodus* but led to a negative search result for the ND3 sequence of *T. emma*. We then performed a pair-wise comparison between the two nucleotide sequences and the ND3 sequences of six additional orthopteran, one coleopteran and three dipteran species. Nucleotide identities in comparison to the six additional orthopteran sequences varied from 69.7 to 88.7% for *T. commodus* and from 48.3 to 50.5% for *T. emma* (Table S2). Similarly, nucleotide identities in comparison to one coleopteran and three dipteran species varied from 69.3 to 71.9% for *T. commodus* and from 48.3 to 50.5% for *T. emma*. To evaluate whether differences in nucleotide sequence translate into protein sequence, we conducted this pair-wise comparison using the translated amino acid (AS) sequences. AS identities in comparison to six additional orthoptera varied from 60.2 to 68.6% for *T. commodus* and from 23.5 to 26.7% for *T. emma* (Table S3). Shared AS identities in comparison to one coleopteran and three dipteran species varied from 59.0 to 61.9% for *T. commodus* and from 19.5 to 22.5% for *T. emma* (AS position 109–112; Table S3). This sequence comparison further revealed a four amino acid insertion for *T. emma* in a highly conserved region of ND3, which is conserved by all other species examined (Figure S3). Similarly, prediction of secondary structure reveals a high degree of consistency across the length of ND3 among all species examined but reveals a structure for *T. emma* deviating from this common pattern (Figure S4).

## Discussion

It is now well-established that the presence of numts in mitochondrial data sets poses a significant problem to correct data interpretation in a wide array of disciplines, ranging from disease studies and DNA barcoding to the analysis of ancient DNA samples [14], [15], [34]. Most prominently, co-amplification of numt sequences has previously led to falsely identifying disease-causing mutations in humans and to the recovery of mtDNA from bones of the Cretaceous period; in these cases, sequences were later identified as human numts [15], [35], [36]. Similarly, our study strongly indicates that the reference sequence of *T. emma* (NC_011823), used for initial primer design and alignment for *de novo* sequencing the mtDNA of *T. commodus*, contains numt sequence. A sliding window analysis identified the ND3 sequence of *T. emma* to deviate in its mutational pattern from the remainder of the sequence. ND3 is a core subunit of the mitochondrial membrane respiratory chain NADH dehydrogenase (Complex I) and hence of fundamental importance of mitochondrial functionality. Owing to their core function, genes involved in respiratory phosphorylation generally show a high degree of consistency between species. Even though ND3 may reveal considerable synonymous differences between species, these differences generally do not translate to protein replacements and subsequently secondary structure so that protein assembly and hence functionality is maintained.

Apart from the region between AS positions 108 and 126 (minus the insertion), the ND3 sequence of *T. emma* does not reveal any resemblance to known ND3 sequence deposited in public data bases (other than to itself), nor does it share any similarity on the nucleotide, AS and structural level to known versions of ND3 in closely and more distantly related insect species. Consequently, our data strongly indicates that in its current state, the mtDNA sequence of *T. emma* contains a numt in place of the ND3 gene.

The unexpected discovery of a numt in our reference sequence further substantiates the constant threat of numts to mtDNA analyses, especially in cases where *de novo* sequencing of mitochondrial genomes serves as the starting point and basis of subsequent studies. Following our strategy, we have successfully eliminated this source of error and sequenced the mitochondrial genome of the Black Field Cricket (*T. commodus*) in the presence of numts. We have combined serial dilution of template DNA with selective amplification of mtDNA exploiting the circular topology of mtDNA and the strand displacement capacity of φ29 polymerases. Two consecutive serial dilutions, one of the template and one of the RCA reaction, allow for a final dilution factor of our template of approximately 10^−6^ in regard to the original DNA extract. It is hence highly improbable that any residual nuDNA (and therefore numt DNA) present in the final template could amplify to sufficient levels to interfere with subsequent sequencing of true mtDNA. It is possible however, that the template for RCA contains residual nuDNA which may be amplified during the reaction (as can be seen in Figure 1 E–F). However, due to the linearity of nuDNA the reaction is expected to lead to only a handful of synthesized numt sequences at most. In contrast, the same process is expected to lead to thousands of new copies in conjunction with circular genomes [37]. Hence, the subsequent dilution of the RCA product leads to the final exclusion of numt sequence while still providing a strong template for mitochondrial amplification at dilution steps 3 to 4 (10^−2^ to 10^−3^).

Despite the existing breadth of strategies introduced to evade the misleading impact of numts, our approach is likely to add value to the molecular tool kit because this strategy relies on only minute amount of tissue and, in contrast to existing methods offers global applicability. Long distance PCR, for example, may prove vulnerable to unintended co-amplification of numts in species revealing long or repetitive numt sequences [7], [21]. Similarly, the dilution of DNA extracts alone may result in DNA templates containing residual nuDNA (and a weakening signal for mtDNA) while low-speed sedimention/separation of mitochondria from cell extracts can prove difficult depending on sample tissue (*e.g.* inseparable or joined mitochondria in muscle tissue or tissues rich in lipids impacting on the density of media used for centrifugation). RNAse-based approaches need to be applied with caution as dual transcription does occur at least in some plants and only transcriptome analysis can ultimately verify the validity of this approach [10]. Hence, ultrahigh speed centrifugation may prove most promising in defeating numt contamination, albeit with the obvious trade-offs of labour-intensity and complexity of this method. However, a major limitation of this method is the requirement of large amounts of tissue required for DNA extraction, which may not always be available depending on the sample and the species studied.

Our strategy does not rely on large quantities of tissue. Ideally, mature female gametes are used as starting material because they generally contain only a single copy of half the chromosomal set but thousands, if not millions, of copies of mtDNA [38]. If female gametes are not available, the strategy can be comfortably applied to tissue samples weighing no more than 1 mg or even less. Preferably, the chosen tissue is rich in mitochondria, *e.g* muscle tissue. If such tissue is not available, our strategy can be applied to any genomic DNA extract, assuming a higher copy number for the mitochondrial than the nuclear genome. As an example, we have successfully applied this strategy to a genomic DNA extract from blood of the Australian Dingo, resulting in a significant selective enrichment of mtDNA (Figure S5).

If applied successfully, our strategy is not vulnerable to accidental co-amplification of pseudogenes as mtDNA and nuDNA are differentially amplified, with the latter being essentially excluded from amplification *per se*. This separation promotes the use of conserved primers generally prone to the co-amplification of numts, which is of particular interest in attempts of *de novo* sequencing mtDNA of species where no sequence information is available to design specific primers. Our method may also prove valuable in cases where sample tissue is limited or DNA extractions resulted in poor/low quality DNA solutions. Applying RCA to these samples will enrich the mtDNA component in the DNA extract improving general template quality. Although our strategy adds an additional amplification step to enrich mtDNA, raising the question of an increase in PCR errors, we failed to detect any incongruities between two independent amplification and sequencing runs in our data set. This is most likely attributable to high fidelity combined with high proofreading activity of φ29 polymerases, reducing the PCR error rate to 1 in 10^6^–10^7^ bases compared to 1 in 10^4^ bases for standard *Taq* polymerases [39], [40]. A limitation of our method, however, may be the dependence on fresh samples as this methodology relies on the circular topology of mtDNA. In old samples, such as ancient DNA samples or DNA extracts from museum specimen, circularity of mtDNA may be lost as DNA degrades over time and mtDNA can be expected to become increasingly nicked.

In conclusion, with very few exceptions, our strategy can be applied globally. It relies on only minute amounts of tissue, is neither labour- nor cost-intensive and does not require specialised equipment. This reaction can be conducted comfortably within two days, starting from a tissue sample and resulting in a DNA solution highly enriched in mtDNA excluding the presence of nuDNA at any meaningful level. This strategy can prove valuable for the amplification of mitochondrial genes or whole genomes in species revealing numts and/or for which no sequence data is available as reference for *de novo* amplification, providing a solid base for subsequent mtDNA-based studies.

## Supporting Information

Figure S1 **Initial amplification and sequencing using a range of conserved and newly designed primers resulted in the co-amplification of numt sequences.** The presence of numt sequences was discovered after alignments revealed multiple mismatches between overlapping fragments (Figure S1A). Fragments revealing consistently a high number of ‘heteroplasmic’ sites were considered numts assuming heterozygosity of the nuclear locus (Figure S1B).
(TIFF)

Figure S2 **Result of sliding window analysis.** Number of segregating sites per 200 bases (step size: 50 bases).
(TIFF)

Figure S3 **Amino acid alignment of 8 orthopteran, one coleopteran and three dipteran species.** The scoring scheme ranges from 0 for the least conserved alignment position, up to 10 for the most conserved alignment position.
(PDF)

Figure S4 **Amino acid alignment of 8 orthopteran, one coleopteran and three dipteran species and secondary structure prediction.** Secondary structure for each sequence is represented by a color. Secondary structure for each sequence is represented by a colour. If a sequence in the alignment has no colours assigned, this means that either there is no DSSP information available, or that no prediction was possible for that sequence.
(PDF)

Figure S5 **Test of general applicability.** Presence of Mitochondrial and Nuclear DNA in Serial Dilution Series' T-DNA I & T-DNA II in genomic DNA extract from blood of the Australian Dingo. T-DNA I was used as template in A–B and T-DNA II in C-D. The highest dilution step in which the presence of nuclear DNA was revealed was step 3 (10^−2^, B) and step 4 (10^−3^) for mtDNA primers (A) on T-DNA I. For T-DNA II, dilution step 1 (10^0^) was the highest dilution showing residual nuclear amplification and step 5 (10^−4^) for mtDNA. Controls (ctrl) are no template controls in A–C and positive control in D. M: DNA ladder (100 bp).
(TIFF)

Table S1 **Primer sequences for the amplification and subsequent sequencing.**
(XLSX)

Table S2 **Pair-wise comparison of ND3 sequences between 8 ensiferan, one coleopteran and three dipteran species.** Values identify the number of identical nucleotides between single pairs [%].
(XLS)

Table S3 **Pair-wise comparison of ND3 sequences between 8 ensiferan, one coleopteran and three dipteran species.** Values identify the number of identical amino acids between single pairs [%].
(XLS)
