# Supplementary figures and images for: Selective Enrichment and Sequencing of Whole Mitochondrial Genomes in the Presence of Nuclear Encoded Mitochondrial Pseudogenes (Numts)
Source: PLoS One. 2012 May 14;7(5):e37142. doi: 10.1371/journal.pone.0037142 (PMC3351421; doi:10.1371/journal.pone.0037142)

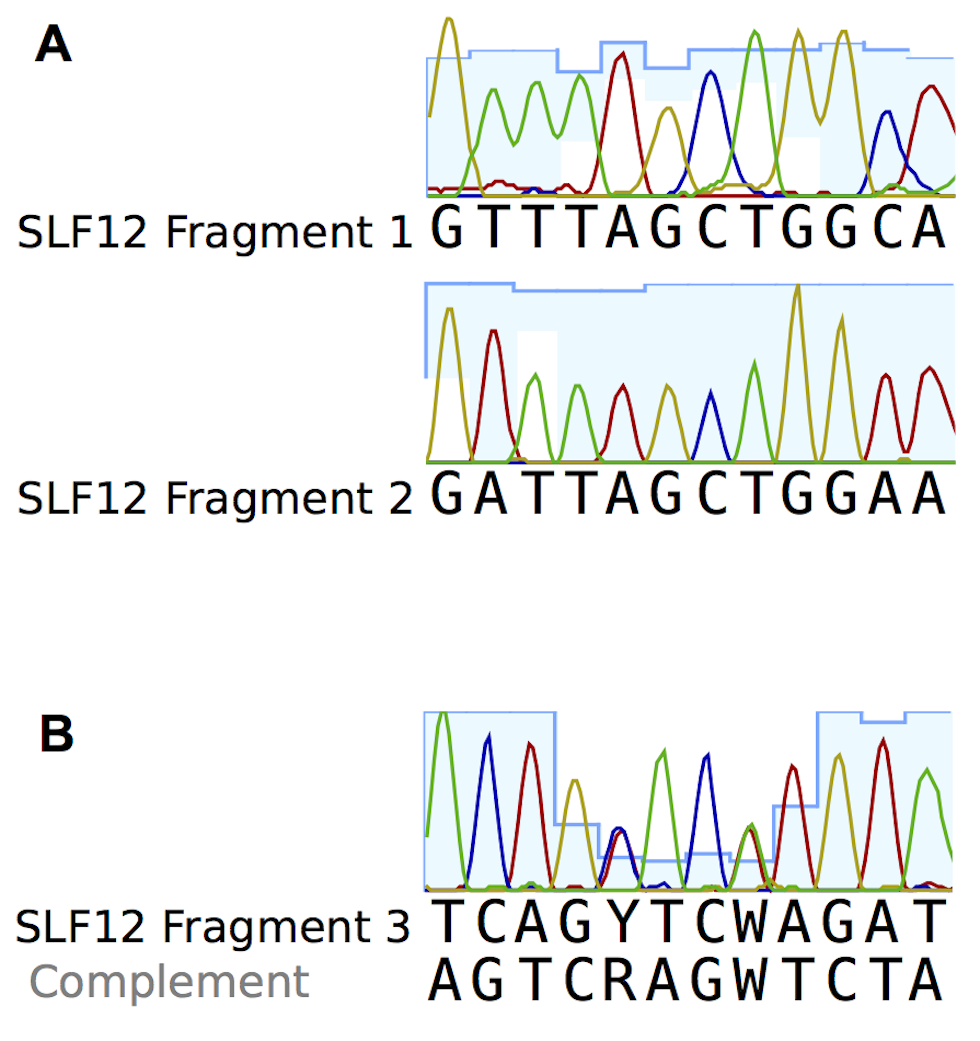

Supplement: Figure S1 — Initial amplification and sequencing using a range of conserved and newly designed primers resulted in the co-amplification of numt sequences. The presence of numt sequences was discovered after alignments revealed multiple mismatches between overlapping fragments (Figure S1A). Fragments revealing consistently a high number of ‘heteroplasmic’ sites were considered numts assuming heterozygosity of the nuclear locus (Figure S1B). (TIFF) [file pone.0037142.s001.tiff]

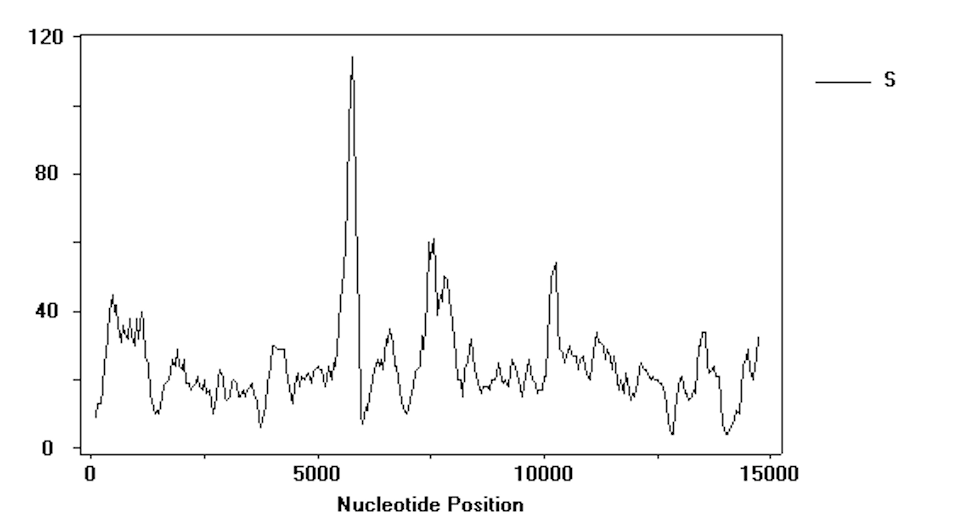

Supplement: Figure S2 — Result of sliding window analysis. Number of segregating sites per 200 bases (step size: 50 bases). (TIFF) [file pone.0037142.s002.tiff]

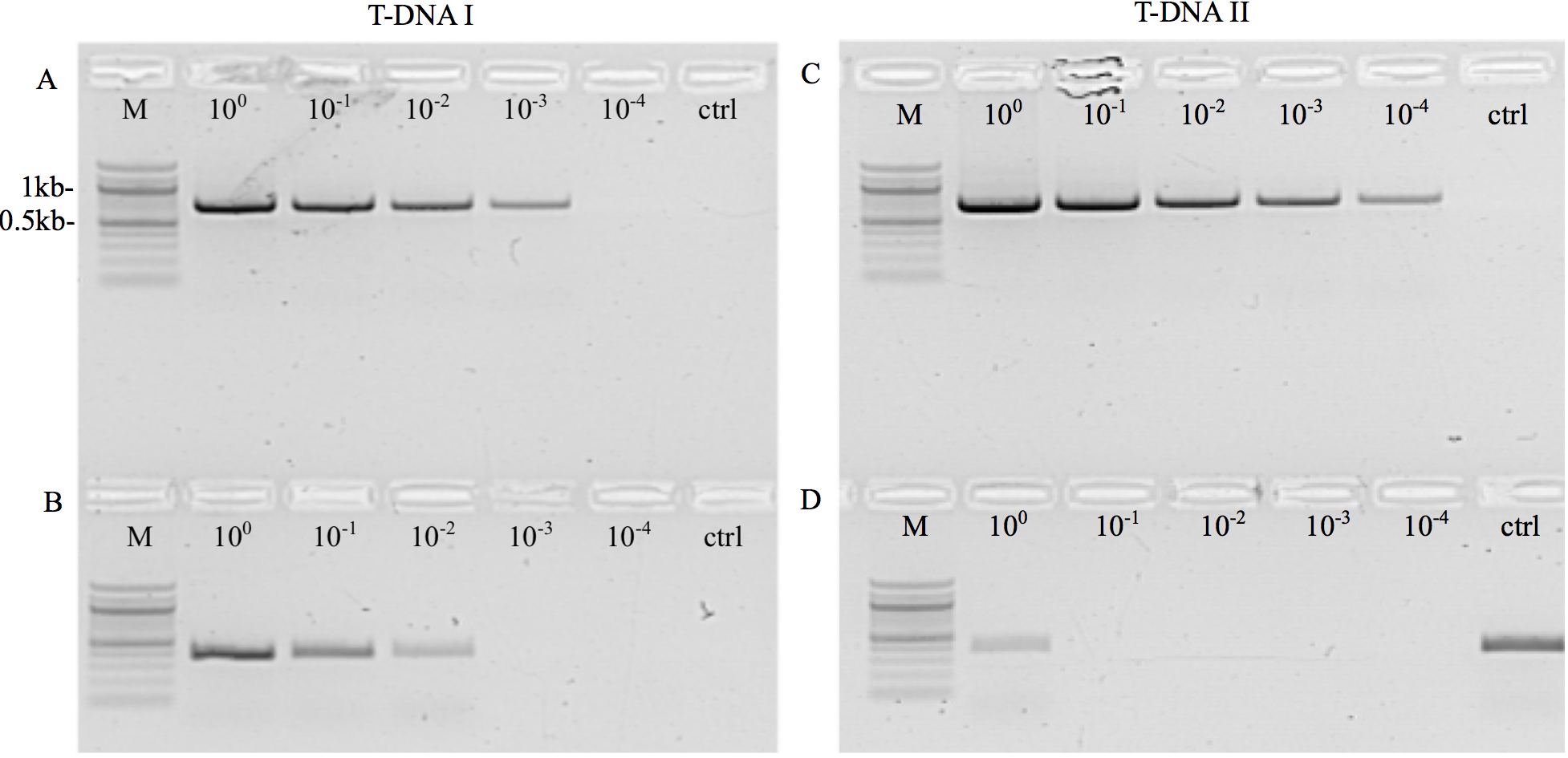

Supplement: Figure S5 — Test of general applicability. Presence of Mitochondrial and Nuclear DNA in Serial Dilution Series' T-DNA I & T-DNA II in genomic DNA extract from blood of the Australian Dingo. T-DNA I was used as template in A–B and T-DNA II in C-D. The highest dilution step in which the presence of nuclear DNA was revealed was step 3 (10−2, B) and step 4 (10−3) for mtDNA primers (A) on T-DNA I. For T-DNA II, dilution step 1 (100) was the highest dilution showing residual nuclear amplification and step 5 (10−4) for mtDNA. Controls (ctrl) are no template controls in A–C and positive control in D. M: DNA ladder (100 bp). (TIFF) [file pone.0037142.s005.tiff]
