# Supplementary material for: Selective Enrichment and Sequencing of Whole Mitochondrial Genomes in the Presence of Nuclear Encoded Mitochondrial Pseudogenes (Numts)
Source: PLoS One. 2012 May 14;7(5):e37142. doi: 10.1371/journal.pone.0037142 (PMC3351421; doi:10.1371/journal.pone.0037142)
Supplement: Figure S3 — Amino acid alignment of 8 orthopteran, one coleopteran and three dipteran species. The scoring scheme ranges from 0 for the least conserved alignment position, up to 10 for the most conserved alignment position. (PDF) [file pone.0037142.s003.pdf]

Results colour-coded for amino acid conservation

The current colourscheme of the alignment is for **amino acid conservation**.

The conservation scoring is performed by PRALINE. The scoring scheme works from 0 for the least conserved alignment position, up to 10 for the most conserved alignment position. The colour assignments are:

Unconserved 0 1 2 3 4 5 6 7 8 9 10 **Conserved**

```
                  .........10 .........20 .........30.........40.........50
Arcyptera_corea   ---MLILSMS IMISFTMPMI VMLIATILSK KII-N-DREK SSPFECGFDP
Teleogryllus_co   --MILIISII ISFTLILSII LMFLSLTL-S KKS-LLDREK ISPFECGFDP
Drosophila_mela   --MFSIIFIA LLILLITTIV MFLASILS-K KAL-I-DREK SSPFECGFDP
Culex_pipiens_p   -MILILLIMS CIIFIITIVV MMLATFLS-K KTI-I-DREK SSPFECGFDP
Phlaeoba_albone   ---MLILSLS ILISFIIPMI VMLIATTLSK KLI-N-DREK SSPFECGFDP
Anopheles_darli   ---MLMLSTM TLIIMIITIV VMILATLLSK KTL-T-DREK CSPFECGFDP
Gastrimargus_ma   ---MMMLMTT IIISFLLPMI IMLMATML-S KKSIN-DWKK GSPFECGFDP
Anoplophora_gla   --MLIIFLLI IMILLIIFIL IIMLNLIS-K KSF-S-DREK SSPFECGFDS
Chorthippus_chi   ---MLTLSTS ILISFTMPMM IMLIATILSK KLI-N-NREK SSPFECGFDP
Thrinchus_schre   --MMTLSVTF MISFLVPMLV MLLATILS-K KII-N-DREK SSPFECGFDP
Oxya_chinensis_   ---MLTLFSS MTICFIIPMI VMLIATMLSK KLI-N-DREK SSPFECGFDP
Teleogryllus_em   MMITSTINYF QPLFYTIIMN TLIINFTKKK DFKMEKNSSH LS-WGIWIQK
Consistency       0016667444 5574665377 6776656518 8450408788 5*89888997

                  .........60 .........70 .........80.........90.........100
Arcyptera_corea   KSSARMPFSL RFFLIAVIFL IFDVEIALIL PIVIIMKTSN IMVWTMSSMF
Teleogryllus_co   KSSARLPFSL RFFLIAVIFL IFDVEIALIM PAMIIQMSSN PLYWIFTLMF
Drosophila_mela   KSSSRLPFSL RFFLITIIFL IFDVEIALIL PMIIIMKYSN IMIWTITSII
Culex_pipiens_p   MNYSRLPFSL RFFLIAIIFL IFDVEIALIL PMILIIKSSN LLNWSITSLF
Phlaeoba_albone   KSSARMPFSL RFFLIAVIFL IFDVEIALIL PIVIIMKTSN IMMWTLSTMF
Anopheles_darli   MNSSRLPFSL RFFLIAIIFL IFDVEIALLL PMIMIIKSSN LINWTITSLF
Gastrimargus_ma   KSSARMPFLI QFFLSAVIFL IFDVEIALIL PILIIMKTSN IMMWTLSTML
Anoplophora_gla   KNSARLPFSL QFFLIAVIFL IFDVEITLLF PLIITLKISN MNYSIIFIF-
Chorthippus_chi   KSPARMPFSL RFFLIAVIFL IFDVEIVMIL PIVIIMKTSN IMMWTMSTMF
Thrinchus_schre   KSSARMPFSL RFFLIAVIFL IFDIEIALIL PIVIIMKSSN LMIWTSATLF
Oxya_chinensis_   KSSARMPFSL RFFLIAVIFL IFDVEIALIL PIVIIMKTSN IFVWTMSTMF
Teleogryllus_em   SGGS----VT DFLLWE--YL SKSSEI---F SCNGGENLRY NSRGQYWWGS
Consistency       7768878878 7*9*78789* 8898**6777 8677767588 6547655565

                  .........110.........120......
Arcyptera_corea   FIMILLLG-- --LYYEWNQG ALQWAE
Teleogryllus_co   FILILLLG-- --LYHEWNQG ALQWAE
Drosophila_mela   FILILLIG-- --LYHEWNQG MLNWSN
Culex_pipiens_p   FIFILLIG-- --LYHEWNQG ALEWNN
Phlaeoba_albone   FIMVLLGG-- --LYHEWNQG ALQWAE
Anopheles_darli   FIFILIVG-- --LYHEWNQG ALEWNE
Gastrimargus_ma   FIILLLIG-- --LYYEWNQG AFKWAK
Anoplophora_gla   FIMILIFG-- --LFHEWNQG ALNWAY
Chorthippus_chi   FILVLLSG-- --LYYEWNQG ALQWAD
Thrinchus_schre   FILVLLGG-- --LYHEWNQG ALQWAE
Oxya_chinensis_   FIIVLLGG-- --LYFEWNQG ALKWAE
Teleogryllus_em   LFPTHYYGGV PPVILEWNQG VLQWAE
Consistency       9958883*00 00986***** 896*76
```
