# Supplementary material for: Selective Enrichment and Sequencing of Whole Mitochondrial Genomes in the Presence of Nuclear Encoded Mitochondrial Pseudogenes (Numts)
Source: PLoS One. 2012 May 14;7(5):e37142. doi: 10.1371/journal.pone.0037142 (PMC3351421; doi:10.1371/journal.pone.0037142)
Supplement: Figure S4 — Amino acid alignment of 8 orthopteran, one coleopteran and three dipteran species and secondary structure prediction. Secondary structure for each sequence is represented by a color. Secondary structure for each sequence is represented by a colour. If a sequence in the alignment has no colours assigned, this means that either there is no DSSP information available, or that no prediction was possible for that sequence. (PDF) [file pone.0037142.s004.pdf]

Results colour-coded for secondary structure

The current colourscheme of the alignment is for **secondary structure type**.

The 3-state (H, E C) secondary structure for each sequence is represented by a colour. If a sequence in the alignment has no colours assigned, this means that either there is no DSSP information available (if this was requested), or that no prediction was possible for that sequence (if this was requested).
The colour assignments are:

HELIX (H) STRAND (E) You have selected to perform secondary structure prediction using **DSSP** (Kabsch and Sander, 1983) and **PSIPRED** (Jones, 1999).

```
                          .........10 .........20 .........30.........40.........50
(PRED) Arcyptera_corea    ---MLILSMS IMISFTMPMI VMLIATILSK KII-N-DREK SSPFECGFDP
(PRED) Teleogryllus_co    --MILIISII ISFTLILSII LMFLSLTL-S KKS-LLDREK ISPFECGFDP
(PRED) Drosophila_mela    --MFSIIFIA LLILLITTIV MFLASILS-K KAL-I-DREK SSPFECGFDP
(PRED) Culex_pipiens_p    -MILILLIMS CIIFIITIVV MMLATFLS-K KTI-I-DREK SSPFECGFDP
(PRED) Phlaeoba_albone    ---MLILSLS ILISFIIPMI VMLIATTLSK KLI-N-DREK SSPFECGFDP
(PRED) Anopheles_darli    ---MLMLSTM TLIIMIITIV VMILATLLSK KTL-T-DREK CSPFECGFDP
(PRED) Gastrimargus_ma    ---MMMLMTT IIISFLLPMI IMLMATML-S KKSIN-DWKK GSPFECGFDP
(PRED) Anoplophora_gla    --MLIIFLLI IMILLIIFIL IIMLNLIS-K KSF-S-DREK SSPFECGFDS
(PRED) Chorthippus_chi    ---MLTLSTS ILISFTMPMM IMLIATILSK KLI-N-NREK SSPFECGFDP
(PRED) Thrinchus_schre    --MMTLSVTF MISFLVPMLV MLLATILS-K KII-N-DREK SSPFECGFDP
(PRED) Oxya_chinensis_    ---MLTLFSS MTICFIIPMI VMLIATMLSK KLI-N-DREK SSPFECGFDP
(PRED) Teleogryllus_em    MMITSTINYF QPLFYTIIMN TLIINFTKKK DFKMEKNSSH LS-WGIWIQK

                          .........60 .........70 .........80.........90.........100
(PRED) Arcyptera_corea    KSSARMPFSL RFFLIAVIFL IFDVEIALIL PIVIIMKTSN IMVWTMSSMF
(PRED) Teleogryllus_co    KSSARLPFSL RFFLIAVIFL IFDVEIALIM PAMIIQMSSN PLYWIFTLMF
(PRED) Drosophila_mela    KSSSRLPFSL RFFLITIIFL IFDVEIALIL PMIIIMKYSN IMIWTITSII
(PRED) Culex_pipiens_p    MNYSRLPFSL RFFLIAIIFL IFDVEIALIL PMILIIKSSN LLNWSITSLF
(PRED) Phlaeoba_albone    KSSARMPFSL RFFLIAVIFL IFDVEIALIL PIVIIMKTSN IMMWTLSTMF
(PRED) Anopheles_darli    MNSSRLPFSL RFFLIAIIFL IFDVEIALLL PMIMIIKSSN LINWTITSLF
(PRED) Gastrimargus_ma    KSSARMPFLI QFFLSAVIFL IFDVEIALIL PILIIMKTSN IMMWTLSTML
(PRED) Anoplophora_gla    KNSARLPFSL QFFLIAVIFL IFDVEITLLF PLIITLKISN MNYSIIFIF-
(PRED) Chorthippus_chi    KSPARMPFSL RFFLIAVIFL IFDVEIVMIL PIVIIMKTSN IMMWTMSTMF
(PRED) Thrinchus_schre    KSSARMPFSL RFFLIAVIFL IFDIEIALIL PIVIIMKSSN LMIWTSATLF
(PRED) Oxya_chinensis_    KSSARMPFSL RFFLIAVIFL IFDVEIALIL PIVIIMKTSN IFVWTMSTMF
(PRED) Teleogryllus_em    SGGS----VT DFLLWE--YL SKSSEI---F SCNGGENLRY NSRGQYWWGS

                          .........110.........120......
(PRED) Arcyptera_corea    FIMILLLG-- --LYYEWNQG ALQWAE
(PRED) Teleogryllus_co    FILILLLG-- --LYHEWNQG ALQWAE
(PRED) Drosophila_mela    FILILLIG-- --LYHEWNQG MLNWSN
(PRED) Culex_pipiens_p    FIFILLIG-- --LYHEWNQG ALEWNN
(PRED) Phlaeoba_albone    FIMVLLGG-- --LYHEWNQG ALQWAE
(PRED) Anopheles_darli    FIFILIVG-- --LYHEWNQG ALEWNE
(PRED) Gastrimargus_ma    FIILLLIG-- --LYYEWNQG AFKWAK
(PRED) Anoplophora_gla    FIMILIFG-- --LFHEWNQG ALNWAY
(PRED) Chorthippus_chi    FILVLLSG-- --LYYEWNQG ALQWAD
(PRED) Thrinchus_schre    FILVLLGG-- --LYHEWNQG ALQWAE
(PRED) Oxya_chinensis_    FIIVLLGG-- --LYFEWNQG ALKWAE
(PRED) Teleogryllus_em    LFPTHYYGGV PPVILEWNQG VLQWAE
```
